# The Pesticide Pyrethroid Metabolite 3-Phenoxybenzoic Acid (3-PBA) Impairs Human Cardiac Na_v_1.5 and Action Potential Depolarization

**DOI:** 10.1007/s12012-026-10212-w

**Published:** 2026-10-25

**Authors:** Michael Ramon de Lima Conceição, Leisiane Pereira Marques, Fabiana da Silva Alcantara, Rafael Leite Tavares de Morais, Vinicius Fogaça-Santos, Diego Jose Belato Orts, João Bosco Pesquero, Diego Santos de Souza, Danilo Roman-Campos

**Affiliations:** 1 Laboratory of Cardiobiology, Department of Biophysics, São Paulo Federal University, São Paulo, Brazil; 2 Center for Research and Molecular Diagnostic of Genetic Diseases, Department of Biophysics, São Paulo Federal University, São Paulo, Brazil; 3 Laboratory of Heart Biophysics, Department of Physiology, Federal University of Sergipe, São Cristóvão, Sergipe Brazil; 4 Laboratory of CardioBiology, Department of Biophysics, Federal University of São Paulo, Botucatu Street, 862, Biological Science Building, 7nd floor, São Paulo, Brazil

**Keywords:** 3-phenoxybenzoic acid, Pyrethroids, Late sodium current, SCN5A, Na_v_1.5, Pesticide

## Abstract

Pyrethroid insecticides (PYRs) are widely used worldwide, and their primary metabolite, 3-phenoxybenzoic acid (3-PBA), serves as a near-ubiquitous biomarker of human exposure to PYRs. While epidemiological data consistently link urinary 3-PBA to increased cardiometabolic risk and coronary heart disease, the underlying electrophysiological mechanisms of its direct potential cardiotoxicity remain unexplored. Parent PYRs are known to exert cardiotoxic effects by enhancing the late sodium current (I_Na-L_) and inducing arrhythmia, but the direct effect of 3-PBA on heart excitability has not been previously investigated. This study demonstrates that 3-PBA interacts with human cardiac Na_v_1.5 expressed in Human Embryonic Kidney (HEK293) cells, and significantly increases I_Na-L_ in a concentration-dependent manner with an EC_50_ of 51.3 ± 5.1 µM. The metabolite does not alter the voltage dependence of activation and inactivation, but it significantly delays the recovery from inactivation of the peak sodium current. In human induced pluripotent stem cell–derived cardiomyocytes (CM-hiPSCs) and adult rat ventricular myocytes, 3-PBA reduced maximal depolarization rate (dV/dt), indicating impaired depolarization dynamics and reduced peak sodium current. At the organ level, acute exposure in isolated rat hearts did not significantly alter standard ECG parameters. Collectively, these findings suggest that 3-PBA is not electrophysiologically inert but directly modulates cardiac sodium channel function, although at supraphysiological concentration. This provides new evidence of the biological activity of 3-PBA and suggests that its cardiotoxic potential may become clinically relevant under conditions of high dose and chronic exposure or in susceptible populations.

**Graphical Abstract:**

**Figure:**
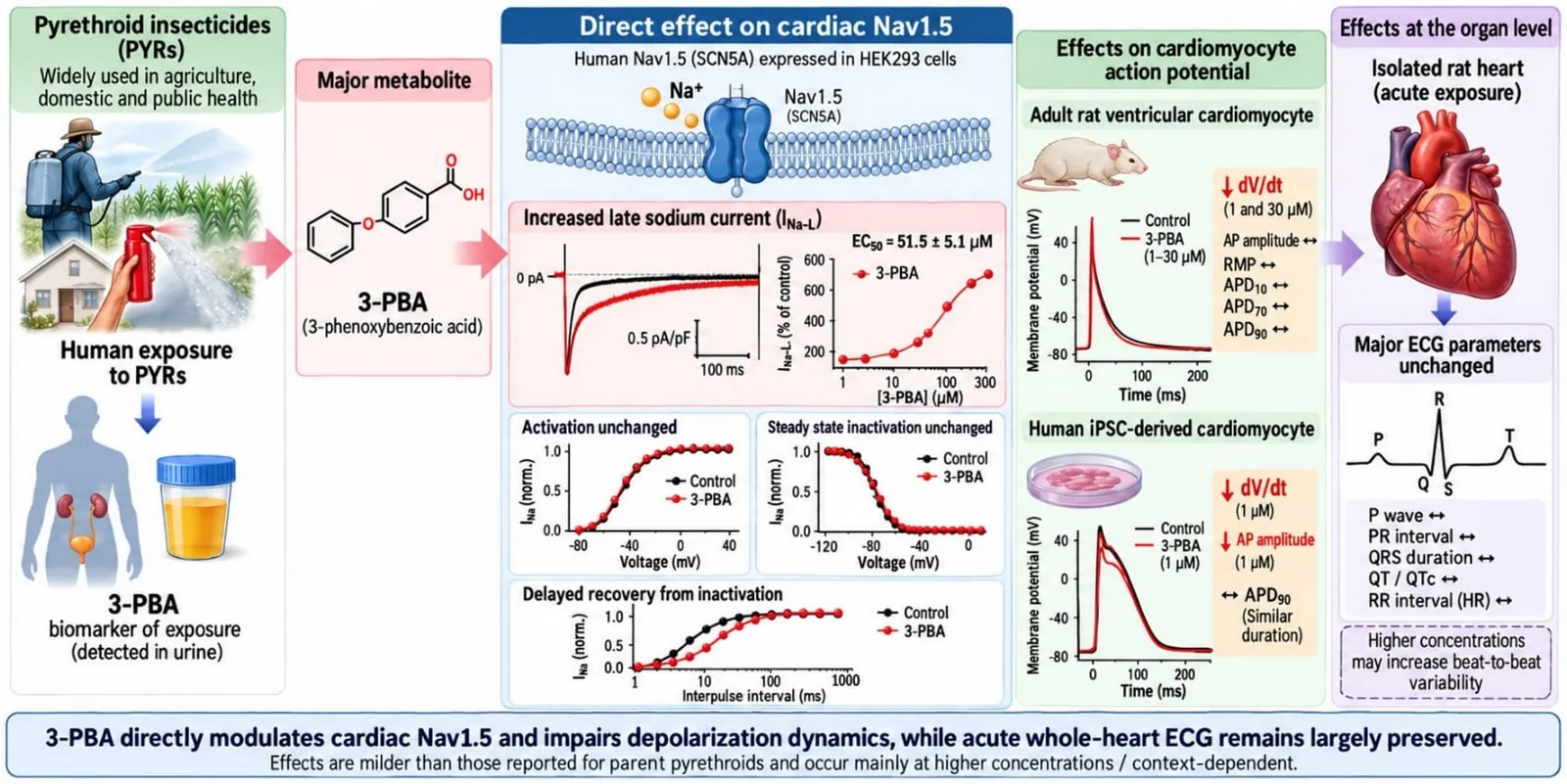

Pyrethroid metabolite 3-phenoxybenzoic acid (3-PBA) increased the late sodium current in Na_v_1.5 expressed in Human embryonic kidney 293 and delayed the recovery from inactivation. Also, 3-PBA in relevant concentration to human toxicology reduced the maximal rate of action potential depolarization (dV/dt) in rat ventricular cardiomyocytes and human induced pluripotent stem cell-derived cardiomyocyte. In the ECG of ex vivo heart preparation 3-PBA 1 μM caused non-effect. This provides evidence of the biological activity of 3-PBA and suggests that its cardiotoxic potential may become clinically relevant under conditions of high dose and chronic exposure or in susceptible populations. Created by Servier Medical Art (https://smart.servier.com), licensed under CC BY 4.0 ativecommons.org/licenses/by/4.0/). Gemini 3.1 Pro was used to refine the layout and visual presentation of the graphical abstract. The authors reviewed, verified, and approved all generated content.

## Introduction

Pyrethroid insecticides (PYRs) account for approximately 25–40% of the global insecticide market and have largely replaced organophosphorus and carbamate compounds in agricultural, domestic, and public health applications due to their high efficacy and predicted low acute and chronic toxicity in mammals [1–3]. Following absorption, PYRs are rapidly metabolized in the liver by carboxylesterases and cytochrome P450 enzymes, producing several metabolites which are predominantly excreted in urine. Among these, 3-phenoxybenzoic acid (3-PBA) is the most common and widely used specific biomarker of PYRs exposure [4–6]. Biomonitoring studies worldwide consistently report near-ubiquitous human exposure to PYRs. 3-PBA is detected in >70% of the general population in North America and Europe, and in >99% of children in certain rural regions of China, with urinary concentrations frequently higher in children than in adults [7–12]. Growing epidemiological evidence links environmental exposure to PYRs, assessed by urinary 3-PBA, with increased cardiometabolic risk. Cross-sectional analyses of the United States National Health and Nutrition Examination Survey (U.S. NHANES, 1999–2014), have shown significant positive associations between urinary 3-PBA concentrations and obesity (particularly in females), diabetes, and prevalence of cardiovascular disease (CVD) and coronary heart disease (CHD) [13–15]. In a nationally representative sample of U.S. adults, individuals in the highest tertile of urinary 3-PBA exhibited 58% higher odds of present CVD (OR 1.58; 95% CI 1.12–2.23) and 75% higher odds of CHD (OR 1.75; 95% CI 1.17–2.61) compared with those in the lowest tertile, with clear dose–response trends [15]. Similar findings have been reported in Chinese populations, where individuals in the highest tertile of 3-PBA showed a markedly elevated risk of developing CHD (OR 3.62; 95% CI 1.48–8.88) [15].

Despite this growing body of epidemiological evidence, the molecular mechanisms underlying the cardiovascular effects of 3-PBA remain unknown. Acute high-dose PYR poisoning has occasionally been linked to transient complete heart block and cardiac conduction disturbances [16]. These acute effects are attributed to the known effect of PYRs in the main voltage-gated sodium channel (VGSC) found in the heart, Na_v_1.5, to disrupt inactivation and lead to a sustained component of sodium current, known as late sodium current (I_Na-L_) [17, 18], a potential arrhythmogenic ion current involved in a range of heart diseases [19, 20].

Although parent PYRs are well-known modulators of VGSC in both the nervous system and cardiac tissue, the potential contribution of their metabolites to cardiovascular toxicity has received limited attention. Emerging experimental evidence suggests that 3-PBA is not biologically inert and may exert cardiotoxic effects. In zebrafish embryo-larval models, 3-PBA induces pericardial oedema, reduces heart rate, inhibits cardiac development, and downregulates ventricular myosin heavy chain (vmhc) gene expression, indicating direct interference with cardiac morphogenesis and function [21]. Furthermore, 3-PBA readily crosses biological barriers, accumulates in tissues, and has been shown to induce dopaminergic neurodegeneration in mammalian models of Parkinson’s disease [22]. Importantly, studies in rats and quail have demonstrated that chronic exposure to PYRs such as permethrin and deltamethrin induces cardiac oxidative stress, inflammation, fibrosis, and upregulation of pro-apoptotic markers. These effects are at least partially independent of direct sodium channel modulation by the parent compounds and may involve contributions from their metabolites [23, 24]**.**

Thus, given that 1—cardiomyocytes express VGSC (primarily Na_v_1.5) essential for action potential (AP) initiation and propagation, 2—the primary target of PYRs are VGSC, 3—considering the structural features of 3-PBA, 4—the repeated epidemiological associations between environmental 3-PBA levels and increased CVD/CHD risk, 5—experimental models suggest potential cardiotoxic effects of 3-PBA, we decided to evaluate whether 3-PBA directly interacts with cardiac sodium channel Na_v_1.5 and modulates AP properties and cardiac excitability.

## Materials and Methods

### Ethical Approval

All procedures involving animals were approved by the Ethics Committee for Animal Use of the Federal University of São Paulo (protocol # 9,781,180,924) and conformed to the Guide for the Care and Use of Laboratory Animals. The experiments were performed using 6–8-week-old male Wistar rats. The animals were housed in standard rodent cages, under a controlled 12-h light/12-h dark cycle at room temperature (23 ± 2 °C) with food and water available ad libitum.

### HEK293 Culture

Human embryonic kidney 293 (HEK293) cells were cultured in DMEM (Dulbecco’s Modified Eagle’s Medium – Invitrogen, Burlington, ON) supplemented with 4.5 g/L of D-( +)-glucose, 10% fetal bovine serum, and 1% of penicillin/streptomycin. Cells were maintained at 37 °C and in a saturated atmosphere with 5% CO_2_. Wild-type human Na_v_1.5 α subunit cDNA, cloned into the BamHI and HindIII restriction sites of the pcDNA 3.1+ vector, was purchased from GenScript Limited (Hong Kong). For transfection, HEK293 cells were plated in 35 mm dishes and grown to 60–80% confluence. Cells were then incubated for 4-5 h at 37 °C and 5% CO_2_ with a transfection mixture containing 800 µL of Opti-MEM (ThermoFisher Scientific), 4 µL of lipofectamine 2000 (Invitrogen), 1 µL of PLUS™ reagent (Invitrogen), and 1 µg of the Na_v_1.5 plasmid DNA. After incubation, the transfection medium was replaced with complete DMEM, and cells were used for experiments 48–72 h post-transfection.

### hiPSC Culture and Cardiomyocytes Differentiation

Human pluripotent stem cells (hiPSCs, ACP5 line, Pluricell) were maintained in 6-well plates coated with 1 mL of GelTrex (Thermo Fisher Scientific) for 30 min. Cells were cultured in StemFlex medium (Thermo Fisher Scientific) supplemented with 10 μM of Rock Inhibitor (RI) (Sigma) for the first 24 h. Subsequently, the medium was replaced with StemFlex alone and refreshed daily. At 70–90% confluence, cells were passaged using Versene (Thermo Fisher Scientific) and replated for differentiation. When cultures reached approximately 80% confluence, cardiomyocyte differentiation was initiated using a Wnt signaling modulation protocol. Briefly, cells were treated for 48 h with 5 µM CHIR99021 (Calbiochem) in RPMI 1640 (Gibco) supplemented with B27 without insulin (RB-medium; Invitrogen) (day 1). The medium was then replaced with RB-medium (day 3), followed by treatment with 2 µM Wnt-C59 (Tocris) in RB-medium for 48 h (day 4). On day 6, cells were maintained in RB-medium for an additional 48 h. On day 8, the medium was switched to RPMI supplemented with B27 containing insulin (RB+ medium) for 72 h, at which point spontaneous contractions were typically observed. Metabolic selection was initiated on day 11 using glucose-depleted RB+ medium. From day 13 onward, cells were maintained in RB+ medium, refreshed every 2 days, until use in electrophysiological experiments. All experiments were performed after day 90 of differentiation. Cardiomyocyte identity (CM-hiPSCs) was confirmed by immunostaining for α-actin, NKX2.5, and cardiac troponin T [25].

### Isolation of Rat Left Ventricular Cardiomyocytes

Left ventricular cardiomyocytes were enzymatically isolated from rats as previously described [26]. Briefly, animals were pretreated with heparin (1000 IU/kg, i.p.) and euthanized 20 min later. Hearts were rapidly excised from the thoracic cavity and placed in ice-cold cell isolation buffer (CIB) supplemented with 0.8 mM EGTA. The aorta was cannulated, and the heart was mounted on a Langendorff perfusion system and perfused at 37 °C. Initial perfusion was performed with CIB containing 0.8 mM EGTA for 10 min. Heart tissue digestion was achieved by perfusion with an EGTA-free CIB solution supplemented with 1 mg/mL type II collagenase, 0.06 mg/mL protease XIV, 0.09 mg/mL trypsin, and 0.3 mM CaCl_2_. After that, the heart was removed from the system, and the left ventricle was dissected, cut into small pieces (~ 2 mm), and further incubated at 37 °C in CIB containing 1 mg/mL type II collagenase, 0.06 mg/mL protease, 0.09 mg/mL trypsin, and 0.7 mM CaCl_2_. The cardiac tissue was then mechanically dissociated using a Pasteur pipette, and the solution was centrifuged at 1,200 rpm for 30 s (Model 206 Baby, Fanem, Brazil). The pellet was resuspended in CIB supplemented with 1.2 mM CaCl_2_ and 2 mg/mL bovine serum albumin (BSA). Cells were centrifuged again (1,200 rpm for 30 s) and finally resuspended in Tyrode’s solution (see below) supplemented with 1 mg/mL BSA for subsequent experiments.

### Electrophysiological Measurements

Macroscopic I_Na_ (HEK293 cells) and action potential (AP) (in rat ventricular cardiomyocytes or CMs-hiPCs) were measured in the whole-cell configuration of the patch-clamp technique, at room temperature (~ 22–25 °C). Glass capillaries were pulled by a Narishige PC-10® stretcher (Narishige© Group, Tokyo, Japan), and the micropipette diameter was approximately 1 µm, with a resistance of 2–3 MΩ. Electronic compensation of capacitance and series resistance (60–70%) was employed. Data acquisition was performed using an EPC-10 USB® patch-clamp amplifier and Patchmaster® software version 9.1 (HEKA Elektronik Dr. Schulze GmbH®, Lambrecht/Pfalz—Germany) or patch-clamp amplifier AxoPatch 200B, a digital interface (Digidata 1550 or 1550A) and Clampex® software version 10.6 (Axon Instruments, Molecular Devices, United States of America). Records were low-pass filtered (cut-off frequency 2 kHz) and digitized at 10–20 kHz.

### Sodium Current

Transfected HEK293 cells were placed on glass coverslips and mounted in the recording chamber. During the experiments, the cells were maintained in an external solution (bath), composed of (in mM): NaCl 130, CaCl_2_ 1.8, CsCl_2_ 5, MgCl_2_ 1.2, HEPES 10, and Glucose 5, pH 7.4 adjusted with CsOH. Internal solution (pipette) was (in mM): Aspartic Acid 50, CaCl_2_ 4.27, CsCl_2_ 60, Na2ATP 5, EGTA 11, MgCl_2_ 1, HEPES 10, pH 7.3 adjusted with CsOH.

I_Na_ was elicited using a square pulse to − 20 mV from a holding potential (V_hold_) of − 120 mV, at a stimulation frequency of 1 Hz. To assess the effect of 3-PBA on the time course of I_Na_, transfected HEK293 cells were exposed to increasing concentrations ranging from 1 to 300 µM. Peak inward sodium current (I_Na-peak_) or I_Na-L_ prior to 3-PBA challenge was considered 100%, which was our control for the concentration–response curve analysis. The percentage of drug effect was determined as the fraction of reduced inward current after the steady-state level following 3-PBA exposition was achieved, normalized by the control inward current. I_Na-L_ was calculated 7 ms after the I_Na-peak_ by a monoexponential decay equation $(y\mathrm{=}{A}_{1 \, }{e}^{-x\mathrm{/}1}\mathrm{+}{y}_{0})$, where ${A}_{1}$ is the amplitude of the decaying component and ${y}_{0}$ is a non-inactivating component. The ${y}_{0}$ was defined as the I_Na-L_. I_Na-L_ after perfusion of increasing concentrations of 3-PBA was expressed as a percentage of the control. Half-maximum effective concentration (EC_50_) was estimated using the equation $y\mathrm{=}{A}_{1 \, }+ \text{ } \frac{({A}_{2}-{A}_{1})}{1\mathrm{+}{10}^{\left({log}_{x0-x \, }\right)p}}$ in which *A*_1_ (bottom asymptote) and *A*_2_ (top asymptote) were fixed at the mean values of the lowest and highest tested concentrations, respectively.

Current–voltage (IV) relationship was recorded by a series of square pulses of 300 ms varying from − 80 mV to +40 mV with an increment of 3 mV every 1 s (1 Hz) from V_hold_ of − 120 mV. Conductance (*G*) was calculated for each membrane potential (${E}_{m}$) according to Ohm’s law as $G\mathrm{=}\frac{I}{\left({E}_{m}-{V}_{rev}\right)}$, where *I* is the amplitude of the current for each test potential and ${V}_{rev}$ is the reversal potential for the Na^+^ determined by the Nernst equation, considering its concentration in the external and internal solution. Conductance was normalized by the maximum conductance (*G*/*G*_max_) for each cell, and the data were fitted using a Boltzmann Eq. (1):

1 $$G\mathrm{=}\frac{{G}_{max}}{{1\mathrm{+}e}^{\left(\frac{{E}_{m-}{V}_{a}}{{k}_{a}}\right)}}$$

Half-maximum activation voltage (${V}_{a}$) and slope factor (${k}_{a}$) were obtained with the equation. Time to peak was defined as the time from the beginning of the pulse to the maximum peak of I_Na_ for each tested potential. Decay time was calculated as the time required for the I_Na-peak_ to decay by 50% of its value at each membrane potential.

Voltage dependence of steady-state inactivation was evaluated by a two-step protocol: 50 ms test pulse to − 20 mV immediately after a 2000 ms conditioning pulse ranging from − 120 mV to − 20 mV with 5 mV increments at each pulse (1 Hz). V_hold_ was maintained at − 120 mV. Peak currents recorded in the test pulse were normalized by the maximum current (I/I_max_) and the data fitted using a Boltzmann Eq. (2):

2 $$\frac{I}{{I}_{max}}\mathrm{=}\frac{1}{{1\mathrm{+}e}^{\left(\frac{{E}_{m}\text{- }{V}_{h}}{{k}_{h}}\right)}}$$

where ${V}_{h}$ and ${k}_{h}$ are the half-maximum inactivation voltage and the curve slope factor, respectively.

Recovery from inactivation was assessed with two square pulses of fixed duration (50 ms and 100 ms, respectively, P1 and P2) to − 20 mV and the variable interval between pulses (1 to 1000 ms). Peak currents at P2 were normalized by the peak currents recorded at P1 (P2/P1). The best fit of the data was obtained using a bi-exponential equation ($y\mathrm{=}{{y}_{0}\mathrm{+}A}_{1 \, }{e}^{-t\mathrm{/}1}\mathrm{+}{A}_{2}{e}^{-t\mathrm{/}2}$). Time constants for the fast (τ1) and slow (τ2) components of the recovery from inactivation were derived from the previously mentioned equation, where *A*_1_ and *A*_2_ represent the maximum recovery of the fast and slow components, respectively.

### Action Potential

Rat left ventricular cardiomyocyte and CM-hiPSCs APs were evoked by a rectangular depolarizing current pulse (3–5 ms in duration, 1 nA) at a stimulation frequency of 1 Hz. During recording, cardiomyocytes were maintained in Tyrode’s solution with the following composition (in mM): 140 NaCl, 5.4 KCl, 0.5 MgCl_2_, 0.33 NaH_2_PO_4_, 11 glucose, 5 HEPES, and 1.8 CaCl_2_ (pH 7.4). For rat left ventricular cardiomyocytes, micropipettes were filled with an internal solution composed of (in mM): 20 KCl, 130 aspartic acid, 130 KOH, 10 HEPES, 2 MgCl_2_, and 5 NaCl (pH 7.2). The internal solution for CM-hiPSCs was composed of (in mM): 140 KCl, 5.0 NaCl, 10 HEPES, 5 Mg-ATP, 5 EGTA (pH 7.2 using KOH). The analyses were performed using Clampfit software (Molecular Devices, v10.7), from which the time to 10, 50, and 90% action potential repolarization (APD10, APD50, and APD90, respectively), maximum rate of depolarization (dV/dt), amplitude (mV), and resting membrane potential (mV) were obtained.

### Electrocardiographic Recordings in Isolated Hearts

Fifteen minutes before heart excision, heparin (1000 IU, i.p.) was administered to prevent coagulation. The hearts were rapidly excised and mounted on a Langendorff apparatus for retrograde aortic perfusion. The preparations were continuously perfused with Krebs–Henseleit solution containing (in mM): 120 NaCl, 5.4 KCl, 1.2 MgCl_2_, 1.8 CaCl_2_, 2 NaH_2_PO_4_, 27 NaHCO_3_, and 11 glucose. The solution was filtered through a 0.45 µm cellulose acetate membrane, equilibrated with a gas mixture of 95% O_2_ and 5% CO_2_, and maintained at 34 ± 0.1 °C, with pH adjusted to 7.4. For ECG acquisition, the isolated heart was kept immersed in a temperature-controlled, water-jacketed chamber containing Krebs solution. Three Ag/AgCl electrodes (1 M NaCl) were positioned within the chamber: one in the right atrium, one at the ventricular apex, and a third serving as a reference electrode. Electrical signals were recorded in lead II configuration using ECG-PC software (version 2.07, TEB – Brazilian Electronic Technology, Belo Horizonte, MG, Brazil) at a paper speed equivalent of 50 mm/s and a gain of 2 N. After a 15-min stabilization period, 1 µM 3-PBA was added to the perfusion solution. Ten minutes later, the solution was replaced with Krebs containing 30 µM 3-PBA. Electrocardiographic parameters, including PR interval, QRS duration, bazett corrected QT interval (QTc), and heart rate (HR) were measured from consecutive beats recorded over 2 min. Data were analyzed offline using Clampfit software.

### 3-PBA Stock Solution

3-PBA (Toronto Research Chemicals (TRC), Canada) was dissolved in DMSO (Fisher Bioreagents, United States) to prepare a stock solution and subsequently diluted in the extracellular recording solution to obtain the final experimental concentrations. The final DMSO concentration was 0.1%.

### Statistics

Data are presented as mean ± SEM (Standard Error Mean). Data analysis and graphing were performed using the Origin Pro® 8 software (OriginLab© Corp., Northampton—USA). The normality of the dependent variable was tested with the Shapiro–Wilk test. Paired t-test was used for comparisons between the two experimental groups. For two or more groups one-way analysis of variance (ANOVA) or linear mixed-effects model, followed by Tukey’s post hoc test, was used, as indicated in the figure captions. Differences were considered statistically significant at p<0.05.

## Results

### 3-PBA Increased I_Na-L,_ and Delayed Recovery from Inactivation of I_Na-peak_

**Fig. 1:**
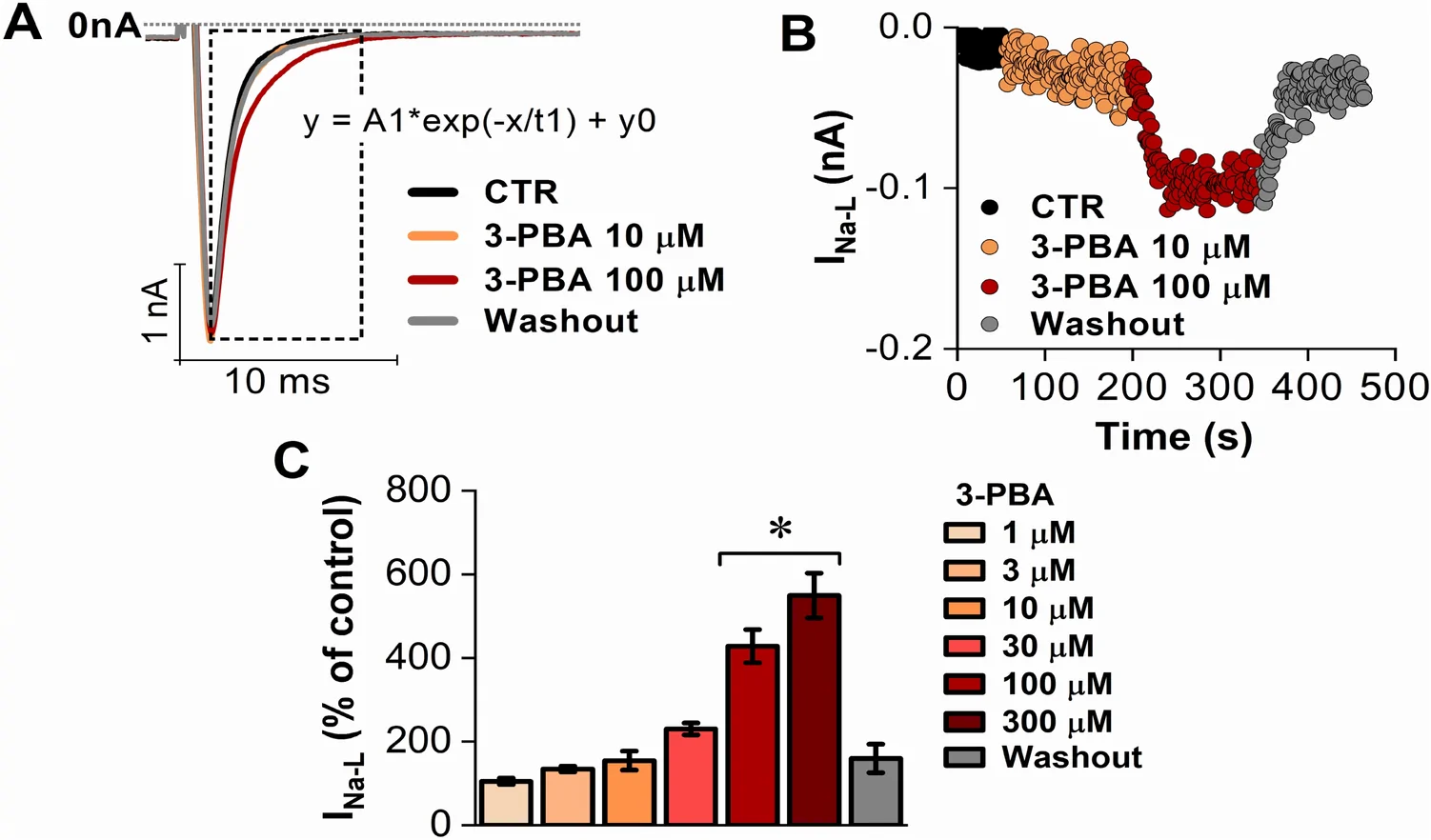
Effect of 3-PBA on the time course of late sodium current (I_Na-L_) of Na_v_1.5 transiently transfected in HEK293 cells. **A** Representative traces of I_Na_ measured in the absence (black) and presence of 10 µM (light red) and 100 µM 3-PBA (dark red). Currents were measured using a square pulse from − 120 mV to − 20 mV at 1 Hz frequency. Dashed square highlights the current used for the exponential adjustment quantification of I_Na-L_. **B** Time course effect of 3-PBA on I_Na-L_ recorded in the absence (black), presence of 10 µM (light red) and 100 µM 3-PBA (dark red), and washout (gray). **C** Effect of increasing concentrations (1 to 300 µM) of 3-PBA (red scale) on the I_Na-L_ expressed as a percentage of control. * Linear mixed-effects model post-test compared to control, p<0.05. n = 4–7 cells per group

I_Na_ was recorded under control conditions (CTR) or in the presence of increasing concentrations of 3-PBA (1 to 300 µM) in HEK293 cells expressing the human isoform of Na_v_1.5. Figure 1A shows representative traces of I_Na_ recorded under control condition (black), in the presence of 10 (orange) and 100 µM 3-PBA (red) and following washout (gray). No significant change in I_Na-peak_ was observed at any concentration tested (data not shown). In contrast, similarly to the PYRs, the 3-PBA metabolite induced an increase in the I_Na-L_ in a concentration-dependent manner (Fig. 1C). The effect was quantified as a percentage relative to I_Na-L_ in the CTR. Compared with CTR, I_Na-L_ augmentation ranged from ~2.3-fold at 30 µM to ~5.5-fold at 300 µM of 3-PBA. The time course of the effect of 3-PBA on I_Na-L_ is shown in Fig. 1B. The effect was almost completely reversed following washout. Half-maximum effective concentration (EC_50_) was estimated in 51.3 ± 5.1 µM. Based on this value, a concentration of 50 µM 3-PBA was selected for subsequent experiments to evaluate its effects on the biophysical properties human Na_v_1.5 transiently expressed in HEK cells.

**Fig. 2:**
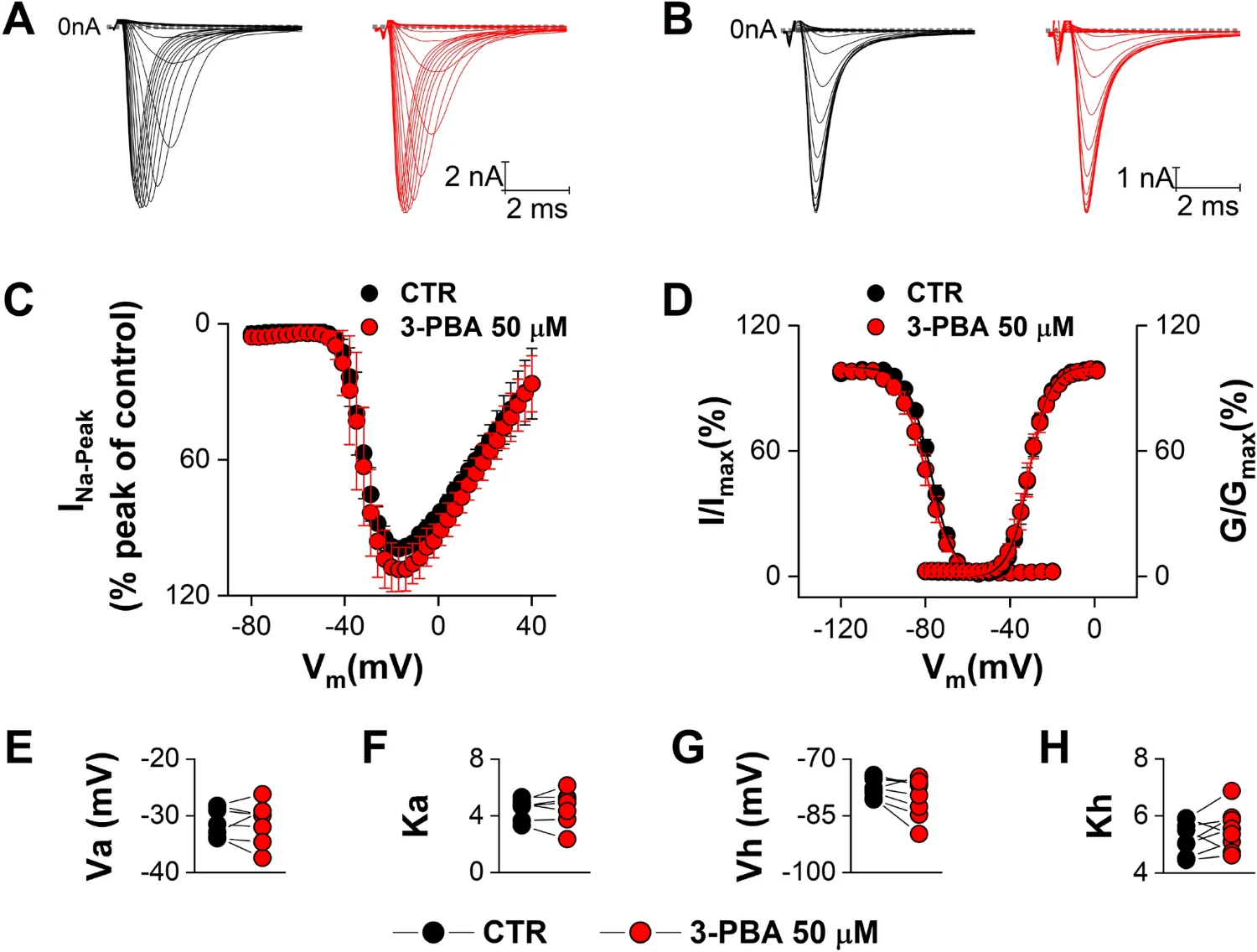
Effect of 3-PBA on the voltage dependence of activation and inactivation of peak sodium current (I_Na-peak_) (Na_v_1.5) transiently transfected in HEK293 cells. **A** and **B** are representative traces of I_Na-peak_ activation, and inactivation measured in the absence (black) and presence of 50 µM of 3-PBA (red) from a V_hold_ of − 120 mV. **C** Current voltage relationship of I_Na-peak_ normalized by the maximum value of I_Na-peak_ in the CTR, in the absence (black) and presence of 50 µM of 3-PBA (red). **D** Conductance normalized by maximal conductance as a function of membrane potential, before (black) and after exposure to 3-PBA (red). Steady-state inactivation curve of I_Na-peak_ after exposure to 50 µM 3-PBA (red) compared to control (black). **E** and **F** correspond to half-maximum (V_a_) and slope factor (K_a_) of activation, respectively. **G** and **H**, correspond to half-maximum (V_h_) and slope factor (K_h_) of inactivation, respectively.* Two-way repeated-measures ANOVA followed by Tukey’s post-hoc test were used to compare I_Na-peak_ across drug conditions over the tested voltage range (**C**). Paired t-test was used to compare the activation and inactivation parameters between control and 3-PBA conditions (E–H), *p<0.05. n = 7–8 cells per group

To assess the impact of 3-PBA on I_Na-peak_, the voltage dependence of I_Na-peak_ activation was studied using a series of square-step depolarization pulses from − 80 mV to +40 mV, from a holding potential of − 120 mV. Exposure to 3-PBA (50 µM) did not cause changes in the activation curve, as illustrated in Fig. 2A and measured in Fig. 2C.

To characterize the effect of 3-PBA on I_Na_, normalized conductance-voltage (G/Gmax) was calculated and is shown in Fig. 2D. Data fitted with a Boltzmann equation revealed no change in the voltage for half-maximal activation (V_a_) (Fig. 2E). Likewise, the slope factor (k_a_) of the activation curve was not altered by 3-PBA either (Fig. 2F). To better understand the possible effect of 3-PBA on Na_v_1.5, we also evaluated the steady-state voltage-dependent inactivation of I_Na-peak_ in the presence of 3-PBA, using a standard two-pulse protocol. Representative current traces are shown in Fig. 2B. As shown in Fig. 2D, exposure to 3-PBA did not change the steady-state inactivation curve of I_Na-peak_, as quantified by voltage for half-maximal inactivation (V_h_) (Fig. 2G) and the slope factor (k_h_) (Fig. 2H).

**Fig. 3:**
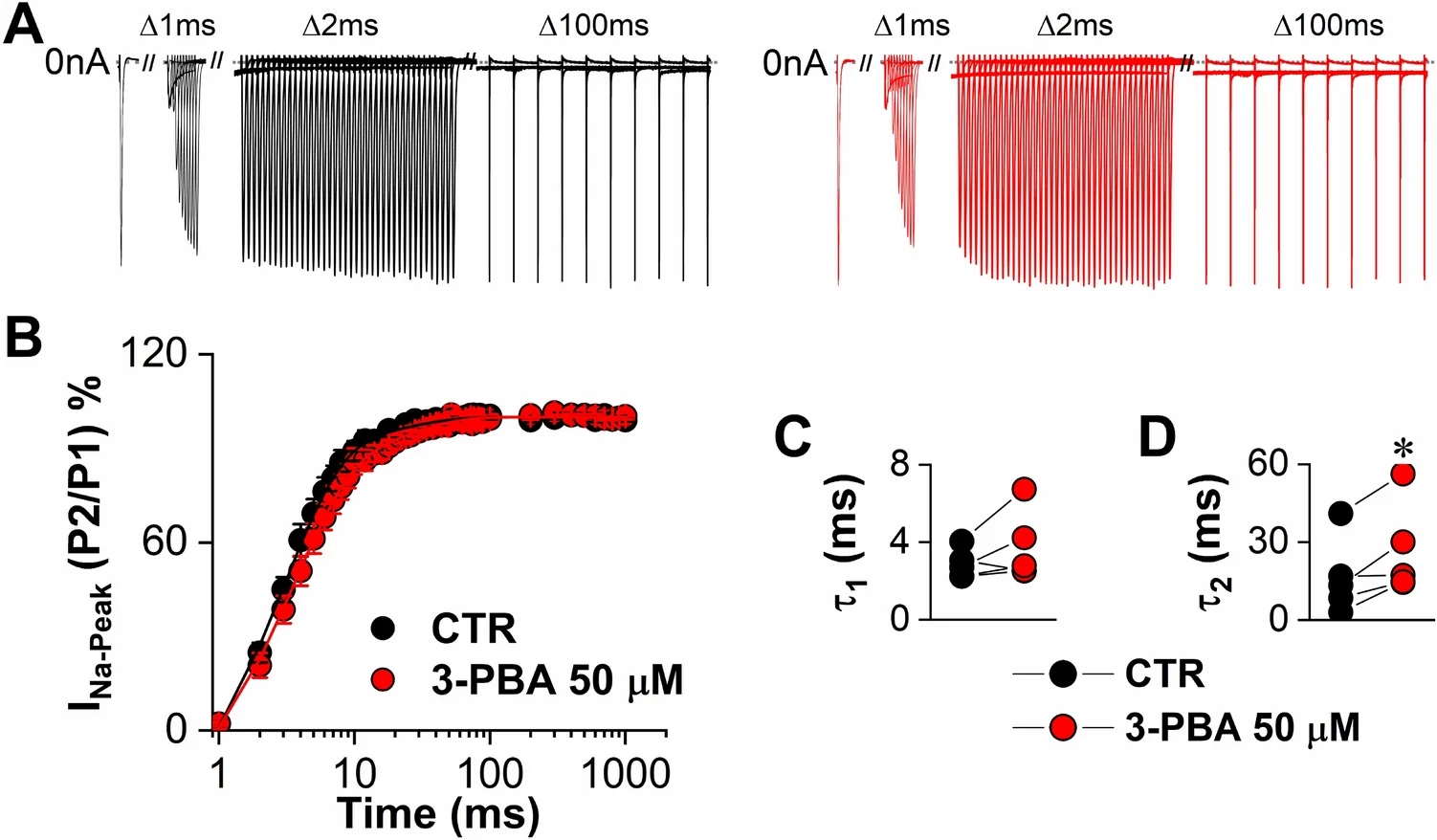
Effect of 3-PBA on the recovery from inactivation of peak sodium current (I_Na-peak_) (Na_v_1.5) (I_Na-peak_) transiently transfected in HEK293 cells. **A** Representative traces of I_Na-peak_ recovery measured in the absence (black) and presence of 50 µM of 3-PBA (red). **B** Normalized I_Na_ (P2/P1) plotted against the interpulse interval. **C** and **D** are the time constant of the fast (τ1) and slow (τ2) components of recovery from I_Na-peak_ inactivation obtained by fitting the data by a bi-exponential equation. *Paired t-test, p<0.05. n = 5 cells per group

To provide additional insights into the ability of 3-PBA to delay I_Na-peak_ decay, we assessed the recovery from I_Na-peak_ inactivation, using a standard two-pulse protocol with increasing inter-pulse intervals. Representative current traces illustrate the recovery kinetics in Fig. 3A. Exposure to 3-PBA slowed I_Na-peak_ recovery time, as evidenced by a slight but significant rightward shift of the recovery curve (Fig. 3B). To quantify the phenomenon, data were fitted with a double-exponential function to obtain the recovery time constants (τ1 and τ2). As is evident in Fig. 3C, D 3-PBA delayed the slow component (τ2).

### Effect of 3-PBA on Action Potential from Rat Left Ventricular and CM-hiPSCs

**Fig. 4:**
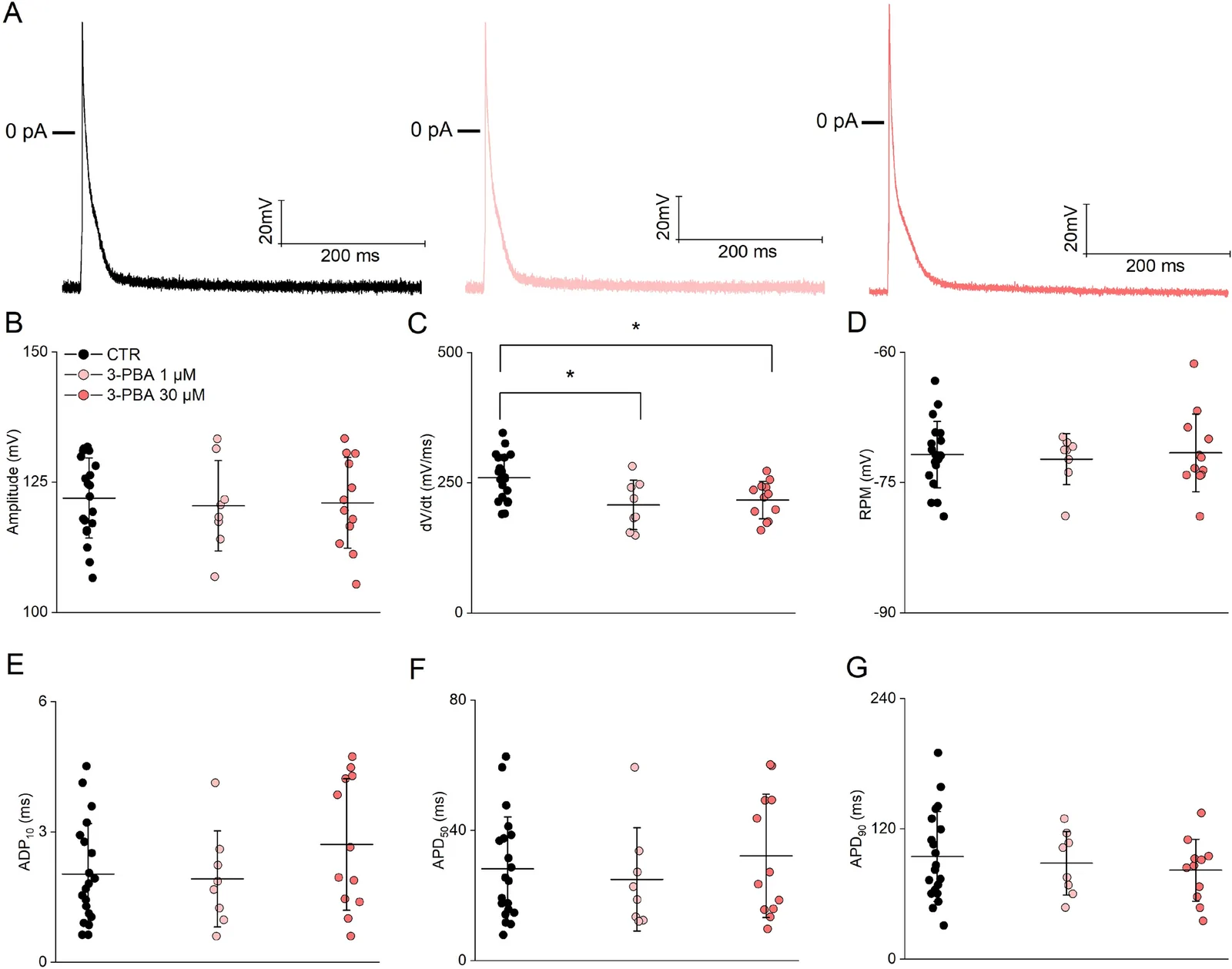
Effect of 3-PBA on rat left ventricular action potentials. **A** Representative action potential traces measured in the absence (black) and presence of 1 µM (light red) and 30 µM of 3-PBA (dark red). **B** Action potential amplitude (mV). **C** maximal velocity of depolarization dV/dt (mV/ms). **D** Resting membrane potential (baseline) (mV). Action potential duration (APD) at 10% (**E**), 50% (**F**) and 90% (**G**) *One-way ANOVA followed by Tukey’s post-hoc test, *p<0.05. n = 8–20 cells per group

To assess the impact of 3-PBA on cellular excitability, APs were recorded in isolated rat left ventricular cardiomyocytes exposed to 1 µM and 30 µM 3-PBA. As illustrated in Fig. 4A, AP amplitude was not significantly altered at either 1 µM or 30 µM compared to control cells (Fig. 4B). Similarly, resting membrane potential (RMP) remained unchanged across groups (Fig. 4D), indicating preserved basal resting membrane voltage stability. In contrast, the maximal upstroke velocity (dV/dt) decreased significantly at 1 and 30 µM (Fig. 4C), showing impairment of depolarization kinetics and possible involvement of sodium channels. Early (APD10%), mid (APD50%), and late (APD90%) repolarization did not differ significantly among groups (Fig. 4E–G).

**Fig. 5:**
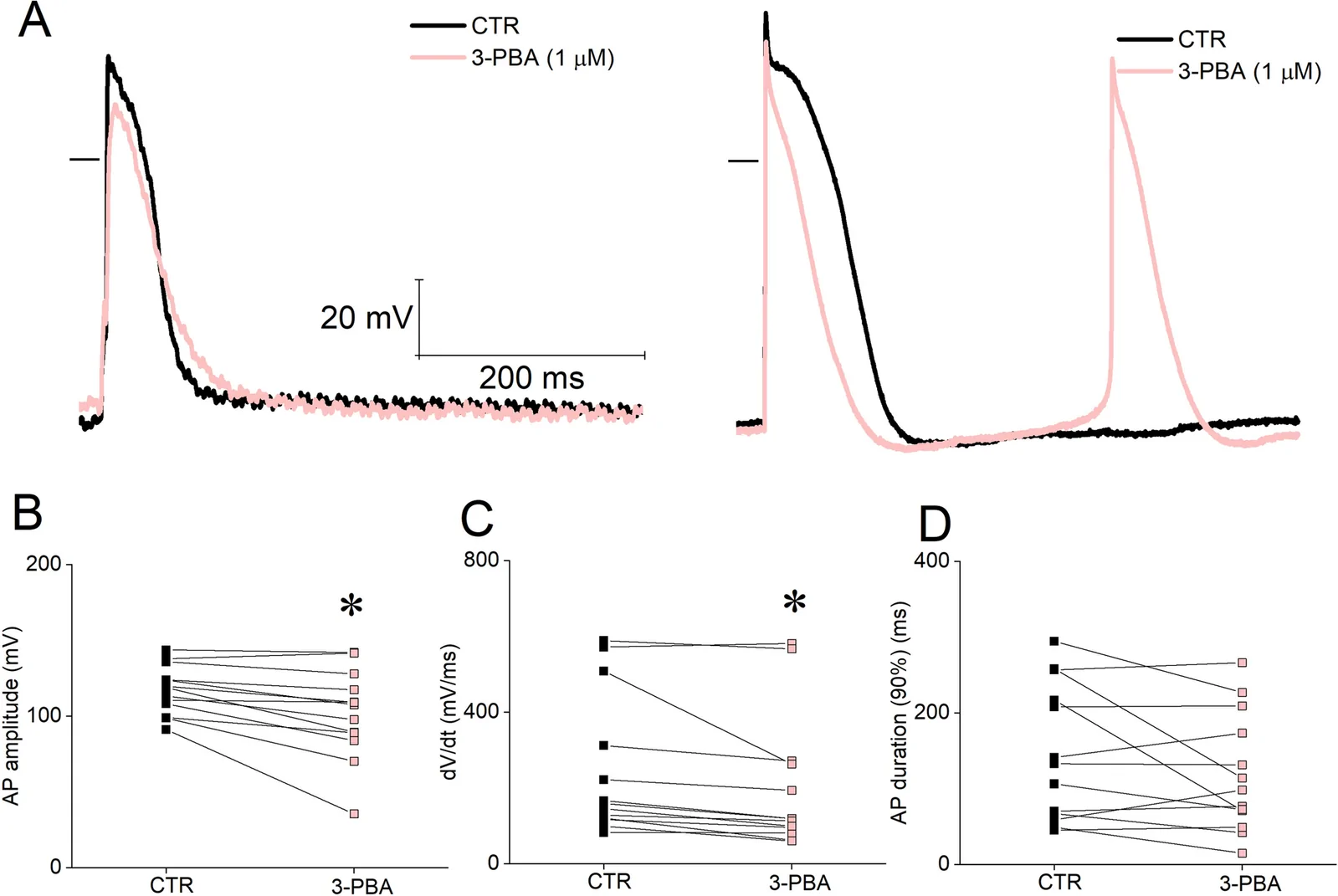
Effect of 3-PBA on action potential from human cardiomyocytes derived from induced pluripotent stem cells (CM-hiPSCs). **A** Representative action potential traces measured in the absence (black) and presence of 1 µM of 3-PBA (light red). **B** Action potential amplitude (mV). **C** maximal rate of action potential depolarization (dV/dt -mV/ms). **D** Action potential duration (APD) at 90% of repolarization. Paired t-test, *p<0.05. n = 13 cells from 3 independent differentiation

To enhance translational relevance, APs were recorded in CM-hiPSCs exposed to 3-PBA, at a concentration within the range relevant to human toxicology. Representative traces of AP from CM-hiPSCs (Fig. 5A) for control conditions and after exposure to 3-PBA (1 µM). From representative traces it is possible to observe significant effects on the APs. Quantitative analysis revealed a significant decrease in AP amplitude compared to control (Fig. 5B). In addition, dV/dt, an indirect index of sodium channel availability, was significantly reduced following 3-PBA exposure (Fig. 5C). In contrast, AP duration at 90% repolarization (APD90) was not significantly altered (Fig. 5D), despite some intercellular variability. Together, these findings indicate that 3-PBA impairs depolarization dynamics in human cardiomyocytes without substantially affecting late repolarization under the experimental conditions tested.

## Electrocardiographic Parameters in Isolated Rat Hearts

**Fig. 6:**
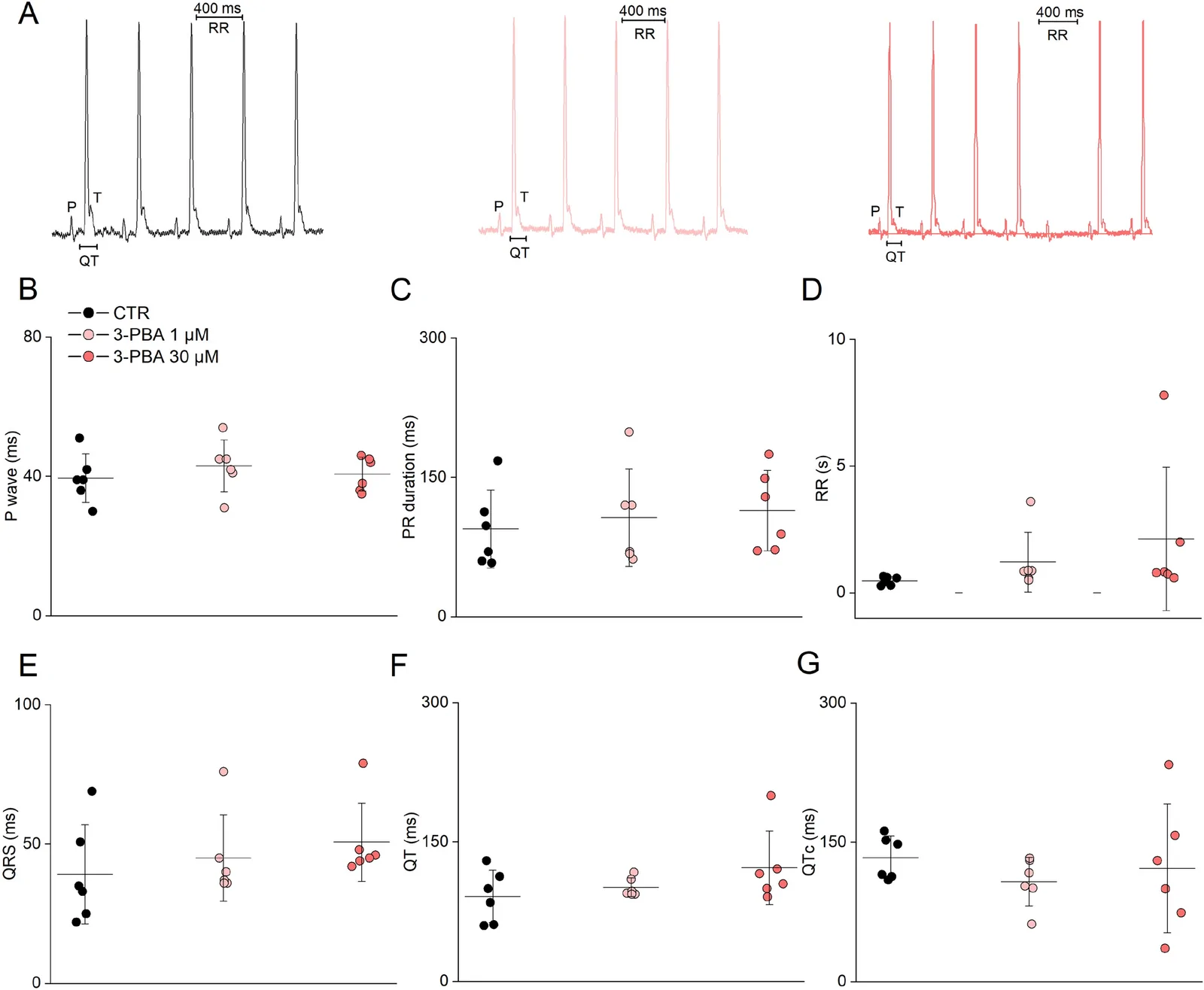
Effect of 3-PBA on ex vivo electrocardiographic parameters. **A** Representative traces of electrocardiographic parameters in the control situation, 3-PBA 1 µM (light red) and 30 µM (dark red). **B** P-wave. **C** PR interval duration. **D** RR interval. **E** QRS. **F** QT. **G** QTc. *One-Way ANOVA followed by Tukey’s post-hoc test, p<0.05. n=6 animals per group

To evaluate the impact of 3-PBA on whole-heart electrophysiological parameters, electrocardiographic recordings were obtained under control conditions and after exposure to 1 µM and 30 µM of 3-PBA (Fig. 6). RR interval analysis revealed no changes in the heart rate. Similarly, P wave duration remained comparable among groups, indicating preserved atrial depolarization. PR interval duration showed no significant differences between control and treated groups, suggesting that atrioventricular conduction was not markedly affected by 3-PBA at the tested concentrations. In addition, QTc interval values did not significantly differ across groups, indicating that global ventricular depolarization–repolarization timing was largely maintained. Overall, these data indicate that acute exposure to 3-PBA does not produce major alterations in healthy ex vivo ECG heart parameters, although higher concentrations may increase beat-to-beat variability without significantly modifying conduction intervals or ventricular repolarization duration.

## Discussion

3-Phenoxybenzoic acid (3-PBA), the major metabolite of PYRs insecticides, has been used as a biomarker of PYRs exposure, which was implicated in various health effects due to widespread environmental exposure. Human biomonitoring studies indicate near-ubiquitous detection of 3-PBA in urine across diverse populations worldwide, with median concentrations ranging from 0.11 to 3.34 μg/g creatinine in environmentally exposed adults and higher levels (0.43–14 μg/g creatinine) in occupationally exposed individuals [27]. Geographic variability has been observed, with higher urinary 3-PBA concentrations reported in Asian countries like China and South Korea compared to North America, often exceeding 1 μg/g creatinine in general populations [28]. Acute exposure to PYRs and their metabolites, including 3-PBA, primarily arises from dietary intake, residential insecticide use, and agricultural applications, leading to rapid metabolism and urinary excretion within hours to days [29].

Regarding the physiological relevance of the experimental conditions, it is noteworthy that the 3-PBA concentration range evaluated in our electrophysiological assays (1 μM to 300 μM) encompasses levels spanning from high-end occupational/accidental poisonings down to environmentally relevant exposures. While background urinary concentrations of 3-PBA in the general population exposed through dietary and ambient sources typically fall within the nanomolar range [7, 30], occupationally exposed agricultural workers and individuals experiencing acute accidental or intentional PYRs poisonings exhibit significantly higher systemic burdens. Blood and tissue levels in such acute or occupational exposure scenarios frequently reach micromolar concentrations, approaching or overlapping with our lowest effective testing doses of 1 μM and 30 μM [31, 32]. Furthermore, because 3-PBA is a lipophilic metabolite that preferentially partitions into lipid-rich cell membranes and cardiac tissue microdomains, local tissue accumulation can result in effective microenvironmental concentrations that far exceed soluble serum levels [33].

Since 3-PBA is usually considered a biomarker of PYRs exposure, in the current investigation we directly evaluated the potential of 3-PBA to modulate mammal heart and cardiomyocyte excitability. Although 3-PBA is traditionally regarded as a relatively inactive metabolite, emerging experimental and epidemiological evidence suggests that it may exert biologically relevant effects, particularly under conditions of repeated or high-level exposure [34]. A recent review by [22] systematically summarized the cardiotoxic effects of PYRs and highlighted that long-term, low-dose exposure may induce oxidative stress, tissue remodeling, and increased susceptibility to arrhythmia, while acute high-dose exposure primarily enhances I_Na-L_. Importantly, the authors also proposed that polymorphisms in the SCN5A gene (e.g., S524Y, S1103Y, R1193Q, V1951L) may modulate individual susceptibility to pyrethroid-induced cardiotoxicity, a concept that may also apply to 3-PBA.

Experimental evidence suggests that 3-PBA can directly interact with cardiac ion channels and modulate heart excitability, while epidemiological studies associate its exposure to broader cardiometabolic risks. Below, we discuss the acute impacts of 3-PBA on the main cardiac sodium channel (Na_v_1.5) and heart excitability, integrating recent experimental findings with existing literature. Importantly, while most available evidence reflects acute exposure scenarios, we cautiously explore potential long-term implications in light of chronic low-level environmental exposure documented in biomonitoring studies [34].

Na_v_1.5, the predominant voltage-gated sodium channel in cardiomyocytes, is critical for AP initiation and propagation, influencing cardiac excitability and conduction [35]. Acute exposure to PYRs is known to prolong sodium channel opening, leading to membrane depolarization and excitability alterations [36], but the role of metabolites like 3-PBA has been less explored. Here, using heterologous expression systems (e.g., HEK293 cells expressing Na_v_1.5), we demonstrate no significant changes in I_Na-peak_ voltage dependence for activation or inactivation with exposure to 50 μM of 3-PBA. In contrast, 3-PBA induced a dose-dependent slowing of channel inactivation, reaching statistical significance at concentrations ≥30 μM, and delayed recovery from inactivation at 50 μM. These effects suggest that 3-PBA may stabilize the open state of Na_v_1.5, potentially prolonging sodium influx during acute high-dose exposures. Although these concentrations exceed typical environmental levels, they may be relevant in accidental or occupational exposure settings. Notably, the EC50 for I_Na-L_ enhancement by 3-PBA (51.3 μM) is considerably higher than the concentrations at which parent PYRs (e.g., β-cyfluthrin) [37] exert potent effects (nanomolar to low micromolar range) [24], consistent with 3-PBA being a less potent sodium channel modulator.

Although 3-PBA did not alter I_Na-peak_ amplitude in HEK293 cells expressing Na_v_1.5, a significant reduction in the maximum upstroke velocity (dV/dt) of the AP was observed in both rat cardiomyocytes and CM-hiPSCs. Also, in CM-hiPSCs, acute exposure to 3-PBA at 1 μM reduces AP amplitude but this effect was not observed in rat ventricular cardiomyocytes even at 30 μM. This discrepancy between the heterologous expression system and functional cardiac cells can be attributed to differences in experimental membrane potentials. I_Na_ measurements in HEK293 cells are recorded from deeply hyperpolarized holding potentials at − 120 mV, ensuring complete removal of inactivation and maximum channel availability [38–40]. Conversely, intact cardiomyocytes maintain a physiological resting membrane potential of approximately − 80 to − 85 mV, at which a substantial fraction of Na_v_1.5 channels resides in a steady-state inactivated condition [41, 42], where xenobiotic exposure can induce a hyperpolarizing shift in inactivation or slow recovery kinetics, causing a cumulative loss of available channels during phase 0 of AP [43, 44].

Furthermore, expression of the isolated Na_v_1.5 alpha-subunit in HEK293 cells lacks the native macromolecular complex present in functional heart tissue. In native cardiomyocytes, Na_v_1.5 dynamically interacts with auxiliary beta-subunits, scaffolding proteins such as ankyrin-G, and lipid microdomains, all of which allosterically modulate channel gating kinetics and pharmacological susceptibility [38, 45]. Importantly, direct quantitative comparisons of I_Na-peak_ amplitude or density among HEK293 cells, hiPSC-CMs, and native ventricular cardiomyocytes are challenged by differences in experimental conditions, including recording temperature, extracellular Na⁺ concentration, current normalization, and the maturation state of hiPSC-CMs. Indeed, substantial variability in reported density and kinetics across cardiomyocyte preparations has been attributed, at least in part, to these methodological differences. Therefore, the mechanistic basis for the differential sensitivity to 3-PBA among these preparations still deserves further investigation.

PYRs, the parent compounds yielding 3-PBA as a metabolite, exert far more potent and multifaceted effects on Na_v_1.5 and cardiac electrophysiology, primarily by binding to distinct receptor sites on the channel’s voltage-sensing domains. This binding stabilizes the open state, markedly slowing inactivation and shifting activation to hyperpolarized potentials, which prolongs sodium influx and I_Na-L_. In contrast to 3-PBA’s restricted impact on recovery and late inactivation at high doses, PYRs like β-cyfluthrin and deltamethrin evoke rapid, concentration-dependent increases in I_Na-L_ even at nanomolar concentrations, leading to AP prolongation, triggered activity (e.g., early/delayed afterdepolarizations), and overt arrhythmia such as ventricular premature beats and torsades de pointes. For instance, β-cyfluthrin disrupts cardiomyocyte contraction, calcium transients, and repolarization in isolated cells, mimicking long QT syndrome effects that are reversible by I_Na-L_ blockers such as ranolazine—phenomena not reproduced by 3-PBA in the present investigation [37].

PYRs also induce resurgent-like tail currents in Na_v_1.5 via interactions with the channel’s β4 subunit C-terminus, promoting "hooked" deactivation kinetics that sustain sodium entry post-repolarization [46]. This contrasts sharply with 3-PBA’s negligible influence on rat ventricular AP repolarization even at 30 μM, underscoring the metabolite’s ~100- to 1,000-fold lower potency. Epidemiological evidence links elevated urinary 3-PBA (a proxy for PYRs exposure) to increased CVD and CHD risk, with odds ratios of 1.58 and 1.75 for the highest vs. lowest tertiles, respectively [16]. However, it is important to interpret these associations cautiously, as urinary 3-PBA likely reflects cumulative PYRs exposure rather than isolated metabolite-driven cardiotoxicity.

The selective AP duration shortening induced by 3-PBA in a subset of CM-hiPSCs, in contrast to its neutral effect on adult rat cardiomyocytes, highlights how the pharmacological actions of 3-PBA interact with model-specific repolarization reserves. As a lipophilic PYRs metabolite, 3-PBA can penetrate the lipid bilayer and can exert allosteric modulations on cardiac ion channels, including subtle reductions in calcium L-type (I_Ca-L_) or I_Na-L_ dynamics [47]. In CM-hiPSCs, the plateau and duration of the AP are uniquely sensitive to minor perturbations in inward currents due to their immature electrophysiological profile, most notably a severe deficiency in the inward rectifier potassium current (I_K1_), encoded by KCNJ2 [46, 48]. Lacking a strong I_K1_ driven stabilizing force, repolarization in CM-hiPSCs is critically balanced between residual I_Ca,L_ and delayed rectifier currents (I_Kr_). Thus, any 3-PBA-mediated suppression of sustaining inward currents (e.g. I_Ca-L_) rapidly triggers premature repolarization, causing a measurable shortening of APD [49]. Conversely, adult rat ventricular myocytes possess an exceptionally robust repolarization machinery dominated by high-density transient outward potassium current (I_to_) and ultra-rapid delayed rectifier potassium currents (I_Kur_), complemented by fully functional I_K1_ [50, 51].

Additionally, the potential contribution of neuronal voltage-gated sodium channel isoforms to 3-PBA-mediated cardiac effects warrants careful consideration. Although Na_v_1.5 tetrodotoxin-resistant, represents the predominant sodium channel subunit in the myocardium, mammalian cardiomyocytes may also express in some cases detectable levels of TTX-sensitive neuronal isoforms, including Na_v_.1, Na_v_1.2, and Na_v_1.6, which are localized preferentially within T-tubules and localized signaling microdomains [52–54]. Given the extensive body of literature demonstrating that 3-PBA and related PYRs derivatives exhibit markedly higher pharmacological affinity for neuronal Na_v_ subtypes compared to cardiac Na_v_1.5 [55, 56], we cannot rule out that in rat cardiomyocyte and iPSC-CMs the observed effect are not only attributed to Na_v_1.5 but could also be related to neuronal isoforms of Na_v_ channels.

This aligns with pyrethroid-induced sodium channel modifications observed in clinical case reports of acute poisoning, where transient cardiac conduction disturbances (e.g., heart block) occur [16]. However, in isolated rat ventricular cardiomyocytes, no AP alterations were noted at 1 or 30 μM, suggesting species-specific differences or compensatory mechanisms in native tissue. Ex vivo rat heart preparations showed no changes in P-wave duration, PR interval, or QT interval with 3-PBA. Due to the robust effect on the heart rate, a larger concentration test was not possible in the isolated murine heart preparation. These observations are consistent with the bradycardia and negative chronotropic effects reported for PYRs such as tetramethrin, which selectively inhibits T-type calcium channels in sinoatrial node cells [57], suggesting that 3-PBA may also affect pacemaker activity through mechanisms beyond Na_v_1.5 modulation.

These findings are consistent with zebrafish embryo studies, in which acute 3-PBA exposure (> 2 mg/L, equivalent to micromolar range) induces bradycardia and abnormal heart formation [21], while lower doses increase heart rate in some models [58], highlighting concentration-dependent biphasic effects on excitability. In rats, acute oral dosing of permethrin yields plasma 3-PBA peaks near 1 μg/mL (≈4.7 μM), correlating with slowed sperm motility but not directly tested for cardiac effects [59]. Extrapolating to long-term exposure, chronic low-level 3-PBA accumulation (e.g., median urinary levels of 0.4–0.7 μg/L in U.S. adults [60]) may subtly impair Na_v_1.5 recovery over time, potentially contributing to arrhythmogenic risks in vulnerable populations, as supported by epidemiological associations between urinary 3-PBA and increased CHD odds (OR=3.62 for the highest tertile) [25]. Longitudinal studies show rising 3-PBA levels in North American children and adults from 2001 to 2010 [61], warranting further investigation into cumulative effects on cardiac excitability.

Metabolically, acute 3-PBA exposure promotes obesity and cardiometabolic diseases, with higher urinary levels (the highest tertile) increasing obesity odds in females (OR=1.22), and is associated with elevated fasting plasma glucose in obese adults [62]. Cross-sectional data link 3-PBA to CVD (OR=1.58 for the highest tertile) and CHD (OR=1.75), potentially via inflammation and oxidative damage [14]. Long-term extrapolation is supported by rising mortality risks (e.g., CVD mortality associated with pyrethroid exposure [14]) and bioaccumulation in food chains [62], emphasizing chronic low-dose effects in high-exposure regions such as in China [63]. A recent study further demonstrated that the fungicide tebuconazole and the herbicide glyphosate can exacerbate pyrethroid-induced cardiotoxicity through oxidative stress and ion channel interactions, suggesting that real-world co-exposures may amplify the risks associated with 3-PBA [64, 65].

In summary, while 3-PBA mirrors PYRs in subtly prolonging inactivation/recovery of I_Na_, its acute effects are milder, delayed, and confined to higher doses without the broad gating disruptions or arrhythmogenic profile of PYRs. Collectively, our findings support the notion that 3-PBA exhibits limited direct electrophysiological toxicity under acute conditions; however, context-dependent effects observed in CM-hiPSCs and whole-heart preparations suggest that its impact may become biologically relevant under sustained exposure, in susceptible individuals, or in combination with other environmental stressors. This perspective refines, rather than fully rejects, the traditional “inactive metabolite” paradigm for 3-PBA and underscores the need for mechanistic and longitudinal studies addressing chronic low-dose exposure scenarios.

## Data Availability

Supporting information available from the corresponding author upon reasonable request.
